# Maleic anhydride-modified chicken ovalbumin as an effective and inexpensive anti-HIV microbicide candidate for prevention of HIV sexual transmission

**DOI:** 10.1186/1742-4690-7-37

**Published:** 2010-04-26

**Authors:** Lin Li, Pengyuan Qiao, Jie Yang, Lu Lu, Suiyi Tan, Hong Lu, Xiujuan Zhang, Xi Chen, Shuguang Wu, Shibo Jiang, Shuwen Liu

**Affiliations:** 1 School of Pharmaceutical Sciences, Southern Medical University, 1838 Guangzhou Avenue North, Guangzhou, Guangdong 510515, China; 2 Lindsley F. Kimball Research Institute, New York Blood Center, 310 East 67th Street, New York, NY 10065, USA

## Abstract

**Background:**

Previous studies have shown that 3-hydroxyphthalic anhydride (HP)-modified bovine milk protein, β-lactoglobulin (β-LG), is a promising microbicide candidate. However, concerns regarding the potential risk of prion contamination in bovine products and carcinogenic potential of phthalate derivatives were raised. Here we sought to replace bovine protein with an animal protein of non-bovine origin and substitute HP with another anhydride for the development of anti-HIV microbicide for preventing HIV sexual transmission.

**Results:**

Maleic anhydride (ML), succinic anhydride (SU) and HP at different conditions and variable pH values were used for modification of proteins. All the anhydrate-modified globulin-like proteins showed potent anti-HIV activity, which is correlated with the percentage of modified lysine and arginine residues in the modified protein. We selected maleic anhydride-modified ovalbumin (ML-OVA) for further study because OVA is easier to obtain than β-LG, and ML is safer than HP. Furthermore, ML-OVA exhibited broad antiviral activities against HIV-1, HIV-2, SHIV and SIV. This modified protein has no or low *in vitro *cytotoxicity to human T cells and vaginal epithelial cells. It is resistant to trypsin hydrolysis, possibly because the lysine and arginine residues in OVA are modified by ML. Mechanism studies suggest that ML-OVA inhibits HIV-1 entry by targeting gp120 on HIV-1 virions and also the CD4 receptor on the host cells.

**Conclusion:**

ML-OVA is a potent HIV fusion/entry inhibitor with the potential to be developed as an effective, safe and inexpensive anti-HIV microbicide.

## Background

Despite extraordinary advances in the development of prevention and therapeutic strategies against human immunodeficiency virus (HIV) infection, HIV/AIDS continues to spread at an alarming rate worldwide. There are approximately 7,400 new infections and over 5,500 new deaths resulting from AIDS each day [1,2]. Unprotected sex is the primary infection route for humans, especially for females, to acquire HIV/AIDS. Therefore, the development of female-controlled topical microbicides is urgently needed [3-5].

An ideal microbicide should be effective, safe, affordable, and easy to use. We previously found that anhydrate-modified bovine proteins, especially 3-hydroxyphthalic anhydride-modified bovine β-lactoglobulin (3HP-β-LG), may fulfill these requirements because they have potent antiviral activities against HIV-1, HIV-2, simian immunodeficiency viruses (SIV) and herpes simplex viruses (HSV). 3HP-β-LG is also effective against some sexually transmitted infection (STI) pathogens, e.g., *Chlamydia trachomatis*. Furthermore, bovine-based proteins are inexpensive, highly stable in aqueous solution, and easy to formulate into topical gel [6-13]. However, since the epidemic of bovine spongiform encephalopathy (BSE) in Europe, serious safety concerns regarding the potential risk of contamination of prion, the pathogen causing BSE, in bovine protein products have been raised. Consequently, the development of bovine protein-based microbicides was discontinued.

Therefore, in the present study, we sought to replace bovine proteins with chemically modified animal proteins of non-bovine origin as new anti-HIV microbicide candidates. All of the non-bovine animal proteins were modified by 3-hydroxyphthalic anhydride (HP), using the same method and the same conditions as 3HP-β-LG. By evaluating the anti-HIV activities of these modifications and the characteristics of proteins used in the reaction, we found that HP-modified chicken ovalbumin (HP-OVA) was the most promising anti-HIV inhibitor among these modified proteins [14]. Since chicken ovalbumin (OVA) is one of the most abundant proteins consumed by people worldwide and is a generally recognized as a safe (GRAS) protein, HP-modified OVA has great potential for further development as an effective, safe and affordable microbicide.

Nonetheless, the phthalate derivatives were reported to have carcinogenic potential [15,16]. Therefore, since HP-OVA may induce a safety concern when used as a microbicide for the prevention of HIV-1 sexual transmission, we searched for new anhydrides to replace HP. To accomplish this, we compared the efficiency of three different anhydrides, including maleic anhydride (ML), succinic anhydride (SU), as well as HP, for the chemical modification of OVA. The relationship of antiviral activities with the percentage of unmodified lysine and arginine in OVA was also investigated. While not as potent as HP-OVA in blocking HIV-1 infection, the safety profiles indicated that ML-OVA may be a more acceptable anti-HIV microbicide candidate. Further mechanism studies showed that ML-OVA could bind both CD4 and gp120 and block HIV-1 envelope glycoprotein (Env) from binding to CD4, indicating that ML-OVA is an effective HIV entry inhibitor. Furthermore, unlike some potent HIV entry inhibitors which are sensitive to trypsin, such as T20 and C34, this modified ovalbumin is resistant to the hydrolysis of trypsin, suggesting that it would also be a stable microbicide when administered to the human vagina.

## Methods

### Reagents

Maleic anhydride (ML), succinic anhydride (SU), 3-hydroxyphthalic anhydride (HP), chicken ovalbumin (OVA, lyophilized powder), rabbit serum albumin (RSA), porcine serum albumin (PSA), bovine serum albumin (BSA), gelatin from cold water fish skin (G-FS), gelatin from porcine skin (G-PS), rabbit anti-OVA serum, FITC-goat-anti-rabbit-IgG, trypsin-agarose beads, phytohemagglutinin (PHA), interleukin-2 (IL-2), XTT [2,3-bis (2-methoxy-4-nitro-5-sulfophenyl)-5-(phenylamino) carbonyl-2H-tetrazolium hydroxide], MTT [3-(4,5-Dimethylthiazol-2-yl)-2,5-diphenyltetrazolium bromide] and 2,4,6-trinitrobenzenesulfonic acid (TNBS) were purchased from Sigma (St. Louis, MO). Calcein-AM was purchased from Molecular Probes Inc. (Eugene, OR). *p*-hydroxyphenylglyoxal (*p*-HPG) was purchased from Fisher Scientific Co. (Valley Park, VA). Recombinant soluble CD4 (sCD4), biotinylated sCD4, gp120 from HIV-1_IIIB_, HIV-1_MN_, and gp105 from HIV-2_ROD _were obtained from Immunodiagnostics Inc. (Woburn, MA). Mouse mAb NC-1 specific for the gp41 six-helix bundle was prepared and characterized as previously described [17]. Seminal fluid (SF) was purchased from Lee. BioSolutions. Inc. (St. Louis, Missouri, MO). Vaginal fluid stimulant (VFS) was prepared as described by Owen and Katz [18].

MT-2 cells, CHO-EE cells, CHO-WT cells, TZM-bl cells, HeLa cells, HeLa-CD4-LTR-β-gal cells, HIV-1_IIIB_-infected H9 cells (H9/HIV-1_IIIB_), U87.CD4.CXCR4 cells, HIV and SIV strains, anti-p24 monoclonal antibody (183-12H-5C), HIV immunoglobulin (HIVIG), pNL4-3 plasmid, pVSV-G plasmid, AZT, AMD3100, Maraviroc, T20, and gp120 from HIV-1_BaL _were obtained from the National Institutes of Health AIDS Research and Reference Reagent Program. Lymphoid cell line CEMX174 5.25M7 expressing CD4 and both coreceptors, CCR5 and CXCR4 [19], kindly provided by Dr. C. Cheng-Mayer, were stably transduced with an HIV-1 long terminal repeat (LTR)-green fluorescent protein (GFP) reporter and LTR-luciferase reporter construct cassette. HSV-2 strain 333 (a low-fusion standard laboratory strain) and Vero cells were generous gifts from Guangzhou Institute of Biomedicine and Health of Chinese Academy of Sciences. VK2/E6E7 cells were purchased from American Type Culture Collection (ATCC) (Manassas, VA). C34 and T20 were synthesized by a standard solid-phase Fmoc (9-fluorenylmethoxy carbonyl) method in the MicroChemistry Laboratory of the New York Blood Center and were purified by HPLC.

### Chemical modification of proteins with different anhydrides under variable conditions

The modified proteins were prepared using a previously described method [6,7,14]. Briefly, non-bovine-origin proteins (RSA, PSA, OVA, G-FS, and G-PS) were dissolved in 0.1 M phosphate (final concentration, 20 mg/ml). 3-hydroxyphthalic anhydride (HP) (final concentration, 40 mM in dimethylformamide) was added in five aliquots in 12 min intervals, while pH was maintained at 8.5. To optimize the conditions for preparation, OVA was treated with 2.5, 5, 10, 20, 40 and 60 mM anhydrides (SU, ML and HP), respectively, or by fixing the concentration of anhydrides in 40 mM and changing the pH values of the reaction system from 3.0 to 10.0. The mixtures were kept for another 1 h at room temperature (RT), then extensively dialyzed against phosphate buffer saline (PBS) and filtered through 0.45 μm syringe filters (Acrodisc; Gelman Sciences, Ann Arbor, MI).

Protein concentrations were determined using the BCA Protein Assay Reagent Kit (Pierce, Rockford, IL). To determine the molecular weights of the modified proteins or macromolecules, SDS-PAGE was used under denaturing conditions. Standard curve, with the log of molecular weight on the Y axis and the relative mobility (R_f_) on the X axis of each standard protein, was plotted. Based on the linear relationship and the R_f _of modified and unmodified proteins, the molecular weights of those modified proteins or macromolecules were calculated.

To quantify lysine residues in modified or unmodified proteins, a TNBS assay was used as previously described [14,20]. Briefly, 25 μl of anhydride modified or unmodified proteins (90 μM) was treated with 25 μl Na_2_B_4_O_7 _(0.1 M) for 5 min at RT. Then 10 μl TNBS were added in the mixture. After another 5 min, 100 μl stop solution (0.1 M NaH_2_PO_4 _and 1.5 mM Na_2_SO_3_) were added to terminate the reaction. The absorbance at 420 nm (*A*_420_) was measured using a microplate reader (Ultra 384; Tecan, Research Triangle Park, NC). The percentage of arginine residues modification was also detected using a previously described method [14,21,22]. In brief, 90 μl of anhydride modified or unmodified proteins (90 μM) in 0.1 M sodium phosphate (pH 9.0) were treated with 10 μl 50 mM *ρ*-HPG for 90 min at RT in the dark. The absorbance at 340 nm (*A*_340_) was measured.

### Detection of inhibitory activity of anhydride-modified OVA on HIV-1 Env-mediated cell-cell fusion

The effect of the three modified OVA proteins on HIV-1 Env-mediated viral fusion/entry was determined using two cell-cell fusion assays [23-25]. In the infectious cell-cell fusion assay, MT-2 cells expressing CD4 and CXCR4 and the infectious H9/HIV-1_IIIB _cells were used as target and effector cells, respectively. Briefly, 1 × 10^4 ^Calcein-AM labeled H9/HIV-1_IIIB _cells were co-cultured with 1 × 10^5 ^MT-2 cells in the presence or absence of modified OVA at graded concentrations at 37°C for 2 h, the fused and unfused Calcein-labeled cells were counted under an inverted fluorescence microscope (Zeiss, Germany). In the non-infectious cell-cell fusion assay, MT-2 cells and the CHO-WT cells that are engineered to express HIV-1 Env as target and effector cells, were used respectively. In brief, 1 × 10^5 ^CHO-WT cells were incubated with 1 × 10^5 ^MT-2 cells in the presence or absence of modified OVA at 37°C for 48 h. Syncytia were counted under an inverted microscope. The percent inhibition of cell fusion and the IC_50 _values were calculated using the Calcusyn software [26].

### Cytotoxicity assay

The *in vitro *cytotoxicity of three anhydride-modified and non-modified OVA to virus target cells (MT-2 and PBMCs) and human vaginal epithelial cells (VK2/E6E7) was measured by the XTT assay. Briefly, 100 μl of modified and non-modified proteins at graded concentrations were added to equal volumes of cells (5 × 10^5^/ml) in wells of 96-well plates. After incubation at 37°C for 4 days, 50 μl of XTT solution (1 mg/ml) containing 0.02 μM of phenazine methosulphate (PMS) were added. After 4 h, the absorbance at 450 nm (*A*_450_) was measured with an ELISA reader. The 50% cytotoxicity concentrations (CC_50_) were calculated using the CalcuSyn software [27].

### Measurement of ML-OVA-mediated antiviral activity

The inhibitory activity of ML-OVA on infection by laboratory-adapted HIV-1 (IIIB, MN and RF) and AZT-resistant strains was determined as previously described [23,28]. In brief, 1 × 10^4 ^MT-2 cells were infected with HIV-1 at 100 TCID_50 _(50% tissue culture infective dose) in the presence or absence of ML-OVA at graded concentrations at 37°C overnight. Then the culture supernatants were changed with fresh medium. On the fourth day post-infection, 100 μl of culture supernatants were collected and mixed with equal volumes of 5% Triton X-100. Then those virus lysates were assayed for p24 antigen by ELISA [23]. Briefly, wells of 96-well polystyrene plates (Immulon 1B, Dynex Technology, Chantilly, VA) were coated with 5 μg/ml HIVIG in 0.85 M carbonate-bicarbonate buffer (pH 9.6) at 4°C overnight, followed by washing with PBS-T buffer (0.01 M PBS containing 0.05% Tween-20) and blocking with PBS containing 1% dry fat-free milk (Bio-Rad Inc., Hercules, CA). Virus lysates were added to the wells and incubated at 37°C for 1 h. After extensive washes, anti-p24 mAb (183-12H-5C), biotin-labeled anti-mouse IgG (Santa Cruz Biotech., Santa Cruz, CA), streptavidin-labeled horseradish peroxidase (SA-HRP) (Zymed, South San Francisco, CA), and 3,3',5,5'-tetramethylbenzidine (TMB) (Sigma) were added sequentially. Reactions were terminated by addition of 1N H_2_SO_4_. Absorbance at 450 nm (*A*_450_) was recorded in a microplate reader (Tecan).

To detect the antiviral activities against T20-resistant strains, HIV-2_ROD_, SHIV_SF33A_, SHIV_SF162P3 _and SIV_mac_251 32H viruses, 100 TCID_50 _viruses were incubated with ML-OVA at graded concentrations at 37°C for 30 min prior to the addition to TZM-bl cells. The culture supernatants were changed with fresh medium 24 h post-infection. At 72 h, the cells were washed and lysed by lysing buffer. Aliquots of cell lysates were transferred to 96-well flat bottom luminometer plates, followed by the addition of luciferase substrate. The luciferase activity was measured in an Ultra 384 luminometer.

The inhibitory activity of ML-OVA on infection by HIV-1_BaL _and primary HIV-1 isolates was determined as previously described [23]. Peripheral blood mononuclear cells (PBMCs) were isolated from the blood of healthy donors at the New York Blood Center by standard density gradient centrifugation by using Histopaque-1077 (Sigma). The cells were plated in 75-cm^2 ^plastic flasks and incubated at 37°C for 2 h. The nonadherent cells were collected and resuspended at 5 × 10^6^/ml in RPMI 1640 medium containing 10% FBS, 5 μg/ml of phytohemagglutinin (PHA), and 100 U/ml of interleukin-2, followed by incubation at 37°C for 3 days. The PHA-stimulated cells (5 × 10^5^/ml) were infected with the corresponding primary HIV-1 isolates at 100 TCID_50 _in the absence or presence of ML-OVA at graded concentrations. Culture media were changed every 3 days. The supernatants were collected 7 days post-infection and tested for p24 antigen by ELISA as described above.

A single-round HIV-1 infection assay was performed using HIV-1_NL4-3 _virions and TZM-bl cells as previously described [5]. Briefly, 1 × 10^4 ^TZM-bl cells were seeded in a 96-well plate and challenged with HIV-1_NL4-3 _(20 ng/well of p24), which were pre-incubated with a chemically modified or non-modified OVA at graded concentrations for 1 h at 37°C. The culture supernatants were replaced with fresh medium 24 h post-infection. The cells were collected 72 h post-infection and the luciferase activity was detected as described above.

To determine the antiviral activity of ML-OVA against herpes simplex virus-2 (HSV-2) infection, HSV-2 at 100 TCID_50 _were incubated with ML-OVA at graded concentrations at 37°C for 30 min prior to the addition to 1 × 10^4 ^Vero cells. After culture at 37°C for 72 h, virus-induced cytopathic effect (CPE) was detected by MTT assay. Briefly, 10 μl of MTT solution (5 mg/ml) was added to each well, followed by incubation at 37°C for 4 h. After the supernatants were removed, 100 μl of DMSO was added, and 5 min later, the absorbance at 570 nm was measured with an ELISA reader (Tecan GeniousPro).

The effective concentration for 50% inhibition (IC_50_) was calculated using the Calcusyn software [26], kindly provided by T. C. Chou (Sloan-Kettering Cancer Center, New York, NY).

### Time-of-addition assay

A time-of-addition assay was performed as previously described [14] to determine the *in vitro *antiviral activity of ML-OVA when added at various time points after virus infection. Briefly, HIV-1_IIIB _(X4 virus) at 100 TCID_50 _was incubated with 1 × 10^5^/ml MT-2 cells for 0, 0.5, 1, 2, 4, 6 and 8 h at 37°C before the addition of ML-OVA (1 μM), AZT (0.1 μM), AMD3100 (0.2 μM) and T20 (0.5 μM), respectively. The culture supernatants were replaced with fresh medium 24 h post-infection. On the fourth day post-infection, the culture supernatants were collected for measuring p24 antigen as described above. The similar procedure was used for testing the inhibitory activity of ML-OVA against HIV-1_BaL _(R5), except that 5 × 10^5^/ml PHA/IL-2-stimulated PBMCs were used, p24 antigen was tested 7 days post-infection, and AMD3100 was replaced by Maraviroc (0.1 μM) as control.

### Assessment of inhibition of ML-OVA on HIV-1 transmission from PBMCs to CEMx174 5.25M7 cells

PHA/IL-2-stimulated PBMCs were isolated and infected by HIV-1_Bal _(a multiplicity of infection of 0.01) for 7 days as described above. After three washes with culture medium to remove free viral particles, 50 μl of HIV-1-infected PBMCs (1 × 10^5^/ml) were incubated with 50 μl of ML-OVA at graded concentration at 37°C for 30 min. Then, 100 μl of CEMx174 5.25M7 cells (2 × 10^5^/ml) were added and co-cultured at 37°C for 3 days. The cells were collected and lysed for analysis of luciferase activity, using a luciferase assay kit (Promega) as described above.

### Trypsin digestion assay

The sensitivity of ML-OVA to digestion by trypsin was tested as described before [29]. Trypsin beads were added to ML-OVA (or the control compounds, T20 or C34) diluted in PBS (final concentration of trypsin = 1 U/ml, ML-OVA = 1 μM, T20 and C34 = 10 μM), followed by incubation at 37°C for different intervals of time (0, 10, 20, 30, 45, 60, 90, 120, 240, 480 and 1,440 min). The supernatants were then collected for detection of the anti-HIV-1_IIIB _activities as described above.

### Detection of the effects of seminal fluid (SF) and vaginal fluid simulant (VFS) on anti-HIV-1 activities of ML-OVA

The effects of human SF or VFS were determined as previously described [30,31]. SF was first centrifuged at 500 g for 30 min to remove spermatozoa. ML-OVA (lyophilized powder) was reconstituted to 550 μM with SF, or VFS, or PBS (control), respectively, followed by an incubation at 37 °C for 60 min. To avoid the toxic effect of SF and VFS on the target cells or viruses, the mixtures were diluted with medium 1000 times (ML-OVA = 0.55 μM) for testing anti-HIV-1_IIIB _activity and 100 times (ML-OVA = 5.5 μM) for testing anti-HIV-1_BaL _activity, respectively, as described above.

### ELISA for detecting the binding of sCD4 with HIV-1 Env

The interaction between sCD4 and the HIV Env proteins was determined as described before [7,14,32]. Briefly, wells of 96-well polystyrene plates were coated with 5 μg/ml HIV-1 Env in 0.1 M Tris buffer (pH 8.8) at 4°C overnight, followed by washing with TS buffer (0.14 M NaCl, 0.01 M Tris, pH 7.0). Then the wells were blocked for 1 h at room temperature with 1 mg/ml bovine serum albumin (BSA) and 0.1 mg/ml gelatin in TS Buffer. Biotinylated sCD4 (1 μg/ml) was pre-incubated with ML-OVA at the indicated concentrations in PBS containing 100 μg/ml BSA for 18 h at 4°C. The mixture, SA-HRP, TMB and 1N H_2_SO_4 _were added sequentially. The *A*_450 _was measured by using an ELISA reader, and the IC_50_values were calculated as described above.

### ELISA for measuring the binding of ML-OVA to monomeric gp120 or sCD4

The binding effect of ML-OVA on monomeric gp120 or sCD4 was determined as previously described [7,32]. Briefly, wells of 96-well plates were coated with 5 μg/ml of gp120 from HIV-1_IIIB _or sCD4 in 0.1 M Tris buffer (pH 8.8) at 4°C overnight, followed by washing with TS buffer. Then the wells were blocked for 1 h at RT with 1 mg/ml BSA and 0.1 mg/ml gelatin in TS buffer. ML-OVA and non-modified OVA at the indicated concentrations in PBS containing 100 μg/ml BSA were added in wells coated with gp120 or sCD4 for 1 h at RT. Rabbit anti-OVA serum, HRP-goat-anti-rabbit IgG (Sigma), TMB and 1N H_2_SO_4 _were added sequentially. The *A*_450 _was measured by using an ELISA reader, and the IC_50 _values were calculated as described above.

### Flow cytometric analysis of the binding of ML-OVA to cells expressing HIV-1 Env or CD4

The binding of ML-OVA with CHO-WT cells that express the HIV-1 Env or HeLa-CD4-LTR-β-gal cells that express CD4 (CHO-EE and HeLa cells bearing neither HIV-1 Env nor CD4 as controls) was determined by flow cytometry as previously described [33,34]. In brief, 100 μl of cells (1 × 10^7^/ml) suspended in PBS contianing 10% goat serum (PBS-GS) were incubated at 4°C for 1 h before addition of 100 μl of ML-OVA (2 μM) or OVA (2 μM). After incubation at 4°C for 1 h, cells were washed three times with PBS-GS. Rabbit anti-OVA serum and FITC-goat-anti-rabbit-IgG were added sequentially. After incubation at 4°C for 1 h, the cells were washed and resuspended in 500 μl of wash buffer, followed by analysis by flow cytometry.

## Results

### Anhydride-modified animal proteins of non-bovine origin were potent inhibitors of HIV-1 infection

Previous studies have shown that bovine milk proteins can be converted into potent inhibitors to prevent sexual transmission of HIV-1 by chemical modification with anhydrides [6,7]. Using a similar approach, we modified five animal proteins of non-bovine origin, including RSA, PSA, OVA, G-FS and G-PS, with a selected acid anhydride, 3-hydroxyphthalic anhydride (HP) and tested their antiviral activities against infections by HIV-1 X4 (HIV-1_IIIB_) and R5 (HIV-1_BaL_) viruses. As shown in Table 1, about 99% of the lysine residues and >93% of the arginine residues in the globulin-like proteins RSA, PSA and OVA were modified by HP, and all of these modified proteins exhibited highly potent antiviral activity against HIV-1 X4 virus, but were less effective against HIV-1 R5 virus. In the two gelatins, G-FS and G-PS, almost 100% of the lysine residues, but only 1-10% of the arginine residues, were chemically modified. Both HP-G-FS and HP-G-PS could also inhibit HIV-1_IIIB _infection activity, but were about 100-fold less potent than HP-modified globulin-like proteins. Neither HP-G-FS nor HP-G-PS could inhibit HIV-1_Bal _infection at the concentration of 8 μM.

**Table 1:** Comparison of the anti-HIV-1 activities and the percentages of modified residues of different compounds modified by 3-hydroxyphthalic anhydride.

HP-modified compounds	% modified residues		Inhibitory activity (μM) on ^a^			
			
			HIV-1_IIIB_		HIV-1_BaL_	
	
	Lysine	Arginine	IC_50_	IC_90_	IC_50_	IC_90_
HP-OVA	99.27 ± 0.60	94.36 ± 1.34	0.006 ± 0.001	0.019 ± 0.005	0.118 ± 0.018	0.359 ± 0.083
HP-RSA	99.00 ± 0.37	92.65 ± 1.23	0.003 ± 0.000	0.006 ± 0.000	0.297 ± 0.036	0.574 ± 0.058
HP-PSA	98.66 ± 0.46	94.31 ± 1.09	0.005 ± 0.001	0.012 ± 0.004	0.411 ± 0.021	0.823 ± 0.030
HP-G-FS	99.63 ± 0.08	1.28 ± 2.21	0.503 ± 0.157	1.268 ± 0.221	>8.00	>8.00
HP-G-PS	99.81 ± 0.09	10.48 ± 1.52	1.182 ± 0.225	3.561 ± 1.314	>8.00	>8.00

^a^Each sample was tested in triplicate, and the experiment was repeated twice.

Although HP-RSA and HP-PSA exhibited anti-HIV-1 activity similar to HP-OVA, we selected HP-OVA for further studies because OVA which is isolated from chicken eggs is much less expensive than RSA and PSA which are purified from animal sera.

### Optimization of experimental conditions for preparation of the most active anhydride-modified ovalbumin

**Figure 1:**
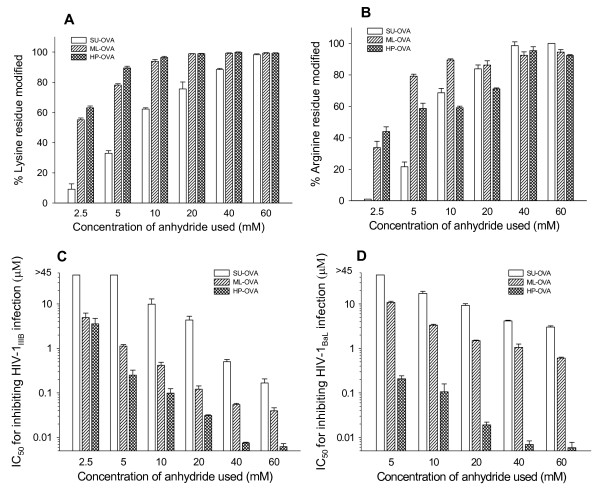
**The effects of anhydride concentrations in the reaction system on the percentages of modified residues and anti-HIV-1 activity of the SU-, ML-, and HP-modified OVA**. The concentration of the anhydrides used is associated with the percentages of modified lysine residues (A) and arginine residues (B) in the chemically modified OVAs and with their anti-HIV-1_IIIB _activity (C) and their anti-HIV-1_BaL _activity (D). Each sample was tested in triplicate, the experiment was repeated twice, and the data are presented in means ± SD.

**Figure 2:**
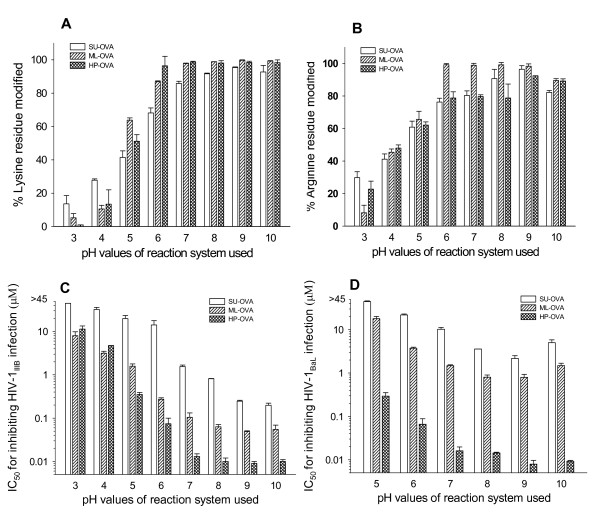
**The effects of pH value in the reaction system on the percentages of modified residues and anti-HIV-1 activity of SU-, ML-, and HP-modified OVA**. The pH value of reaction systems is correlated with the percentages of modified lysine residues (A) and arginine residues (B) in the chemically modified OVAs and with their anti-HIV-1_IIIB _activity (C) and their anti-HIV-1_BaL _activity (D). Each sample was tested in triplicate, the experiment was repeated twice, and the data are presented in means ± SD.

To search for alternate anhydrides to replace 3-hydroxyphthalic anhydride (HP) for modifying OVA, two other anhydrides, maleic anhydride (ML) and succinic anhydride (SU) were used. To optimize the experimental conditions for production of anhydride-modified ovalbumin, we compared the efficacy of SU, ML and HP at different concentrations (2.5, 5, 10, 20, 40 and 60 mM). With the increasing concentrations of anhydrides used, the percentages of the modified lysine and arginine residues increased, reaching a plateau when 40 mM of the anhydrides were used (Fig. 1A and 1B). Then, the possible effect of pH value on the modifications of the lysine and arginine residues in OVA was evaluated by using a fixed concentration (40 mM) of anhydrides under variable reaction system pH values (3.0~10.0). As shown in Fig. 2A and 2B, the percentages of the modified lysine and arginine residues in the modified OVA increased with the increasing pH value of the reaction system. A plateau was reached when the pH was over 8.0.

Based on these results, the average pH of 8.5 and 40 mM of anhydrite were selected as the optimal parameters in subsequent experiments. Under these optimal experimental conditions, the average molecular weights of ML-OVA, SU-OVA and HP-OVA were 45.59, 44.58 and 44.58 kd, respectively, as determined by SDS-PAGE. In addition, 99.19%, 88.40% and 99.86% of the lysine residues and 92.46%, 98.58% and 89.26% of the arginine residues were modified by ML, SU and HP, respectively.

Notably, the percentages of the modified lysine and arginine residues appear correlated with the anti-HIV-1_IIIB _(Fig. 1C and 2C) and anti-HIV-1_BaL _(Fig. 1D and 2D) activity of these modified OVA. Both ML-OVA and HP-OVA with higher percentages of modified lysine and arginine residues had more potent anti-HIV-1 activity than SU-OVA. Similar results were seen in the effectiveness on HIV Env-induced cell-cell fusion (Table 2).

**Table 2:** Inhibitory activity of modified OVA on HIV-1-mediated cell-cell fusion^a^.

Modified OVAs	Anhydride	Fusion by MT-2 & CHO-WT		Fusion by MT-2 & H9/HIV-1_IIIB_	
		
		IC_50 _(μM)	IC_90 _(μM)	IC_50 _(μM)	IC_90 _(μM)
ML-OVA		0.193 ± 0.003	0.789 ± 0.186	0.411 ± 0.090	1.021 ± 0.222
SU-OVA		0.406 ± 0.047	1.986 ± 0.091	1.462 ± 0.142	3.338 ± 0.326
HP-OVA		0.186 ± 0.004	0.386 ± 0.006	0.057 ± 0.005	0.135 ± 0.007
OVA		>100	>100	>100	>100

^a^The measurements were performed in triplicate, and the experiment was repeated twice. Data are presented in means ± SD.

The cytotoxicity of these three modified OVA and unmodified OVA proteins was determined using MT-2, PBMC and VK2/E6E7 cells. As shown in Table 3, the cytotoxicities of ML-OVA and HP-OVA to MT-2, PBMC and VK2/E6E7 cells were about one- and 3-fold higher than that of unmodified OVA, respectively, suggesting that HP-modified proteins exhibit higher cytotoxicity than ML-modified proteins.

**Table 3:** *In vitro *cytotoxicity of anhydrate-modified OVA^a^.

Modified OVAs	MT-2		PBMC		VK2/E6E7	
	
	CC_50 _(μM)	CC_90 _(μM)	CC_50 _(μM)	CC_90 _(μM)	CC_50 _(μM)	CC_90 _(μM)
ML-OVA	187.33 ± 2.329	465.18 ± 34.16	148.29 ± 14.51	447.33 ± 84.30	140.49 ± 6.840	501.60 ± 35.96
SU-OVA	270.93 ± 6.838	540.69 ± 12.60	161.84 ± 6.446	927.39 ± 74.16	188.84 ± 52.69	480.76 ± 240.94
HP-OVA	99.18 ± 3.095	256.14 ± 10.58	90.28 ± 4.113	414.22 ± 52.99	78.39 ± 1.760	331.02 ± 16.44
OVA	340.34 ± 43.22	938.72 ± 513.41	357.20 ± 58.06	896.26 ± 309.08	253.09 ± 74.92	904.94 ± 795.89

^a^Each sample was tested in triplicate, and the experiment was repeated twice.

Though HP-OVA was found to be the most potent modified OVA, we selected the second most effective one, ML-OVA, for further study because of the concerns over the possibility that HP-modified proteins might generate some phthalate derivatives with carcinogenic potential [35-38]. In addition, HP-OVA displayed higher cytotoxicity than ML-OVA (Table 3).

### ML-OVA exhibited potent inhibitory activity against infection by HIV-1, HIV-2, SIV, SHIV and HSV-2 strains

The inhibitory activities of ML-OVA against virus infection were tested on HIV-1, HIV-2, SIV, SHIV and HSV-2 strains. As shown in Table 4, ML-OVA exhibited highly potent inhibitory activity against infection by the laboratory-adapted HIV-1 X4 and X4R5 strains with IC_50 _at nM levels, while it inhibited infection by laboratory-adapted and primary HIV-1 R5 strain with IC_50 _at low μM level. Notably, it was also effective against HIV-1 variants resistant to AZT, a reverse transcriptase inhibitor, and enfuvirtide, an HIV fusion/entry inhibitor, with IC_50 _at nM level. Interestingly, ML-OVA could also inhibit infection by HIV-2, SIV, SHIV and HSV-2 strains, although the IC_50 _values on HIV-2 and HSV-2 were relatively high. These results suggest that ML-OVA displays broad and potent antiviral activities against HIV and SIV.

**Table 4:** Antiviral activities of ML-OVA against infection by HIV-1, HIV-2, SHIV, SIV and HSV-2 strains.

Virus strain	Inhibitory activity (Mean ± SD, μM)^a^	
	
	IC_50_	IC_90_
**Laboratory-adapted HIV-1 strains**		
IIIB (X4)	0.023 ± 0.004	0.057 ± 0.004
MN (X4)	0.151 ± 0.008	0.821 ± 0.103
RF (X4R5)	0.034 ± 0.005	0.147 ± 0.059
BaL (R5)	0.690 ± 0.109	2.236 ± 1.184
**Primary HIV-1 strains**		
UG94103(clade A, X4R5)	0.561 ± 0.159	2.520 ± 1.231
92US657 (clade B, R5)	2.107 ± 0.263	9.357 ± 1.939
93IN101 (clade C, R5)	1.208 ± 0.535	4.946 ± 0.632
BCF02 (clade O, R5)	0.067 ± 0.004	0.188 ± .0.010
Ru570 (clade G, R5)	4.299 ± 0.298	9.332 ± 0.594
**Drug-resistant HIV-1**		
AZT-R ^b^	0.296 ± 0.052	1.099 ± 0.773
NL4-3_D36G_^c^	0.104 ± 0.019	0.591 ± 0.059
NL4-3_(36G)V38A _^c^	0.185 ± 0.027	0.543 ± 0.026
NL4-3_(36G)V38E/N42T _^c^	0.156 ± 0.082	0.992 ± 0.438
**HIV-2**		
ROD	6.079 ± 1.907	12.59 ± 3.022
**SIV**		
mac 251 32H	0.307 ± 0.174	6.128 ± 2.078
**SHIV**		
SF33A (X4)	0.189 ± 0.103	1.505 ± 0.872
SF162P3 (R5)	1.312 ± 0.688	11.63 ± 5.477
**HSV**		
HSV-2 strain 333	39.06 ± 2.316	69.49 ± 3.145

^a^The measurements were performed in triplicate, and the experiment was repeated at least twice;

^b^RTI-resistant strain;

^c ^Enfuvirtide-resistant variants.

### ML-OVA inhibited transmission of cell-associated HIV-1BaL virus from PBMCs to CEMx174 5.25M7 cells

**Figure 3:**
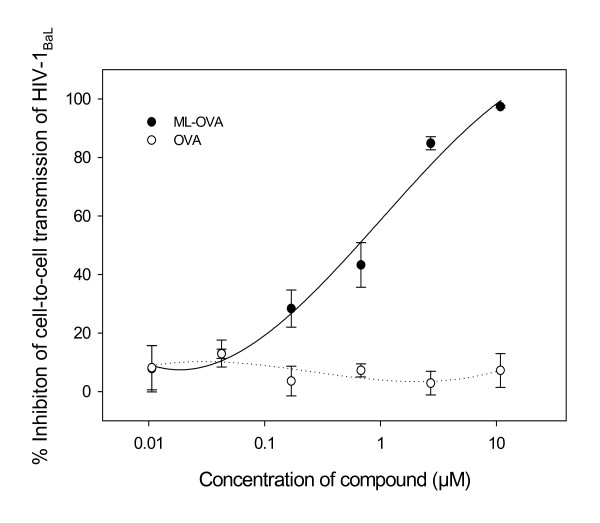
**ML-OVA-mediated inhibition of transmission of HIV-1_BaL _from PBMCs to CEMx174 5.25M7 cells**. All the samples were tested in triplicate, the experiment was repeated twice, and the data are presented in means ± SD.

To determine whether ML-OVA could inhibit HIV-1_BaL _transmission from PBMCs to CEMx174 5.25M7 cells, PBMCs infected by HIV-1_BaL _were cocultured with CEMx174 5.25M7 cells in the presence of ML-OVA at graded concentrations. After 3 days, the level of luciferase activity, representing HIV-1 infectivity in CEMx174 5.25M7 cells, was measured. As shown in Fig. 3, ML-OVA blocked transmission of HIV-1_BaL _from PBMCs to CEMx174 5.25M7 cells, suggesting that it can prevent transmission of cell-associated HIV-1 isolates.

### ML-OVA exerted its antiviral action at the early stage of HIV-1 replication

**Figure 4:**
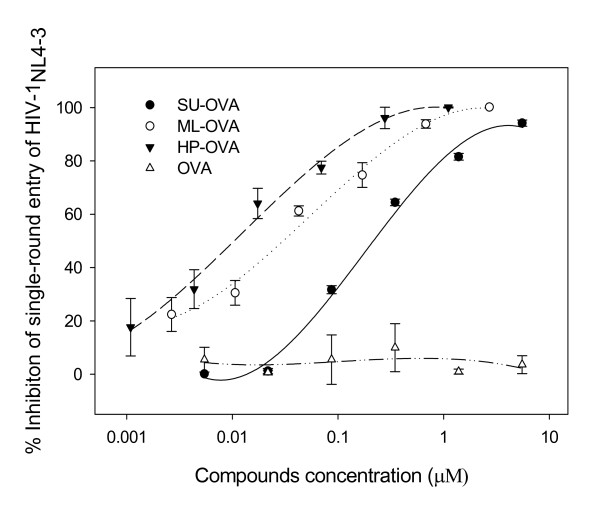
**Inhibition of chemically modified OVA on single round entry of HIV-1_NL4-3_**. Each sample was tested in triplicate, the experiment was repeated twice, and the data are presented in means ± SD.

**Figure 5:**
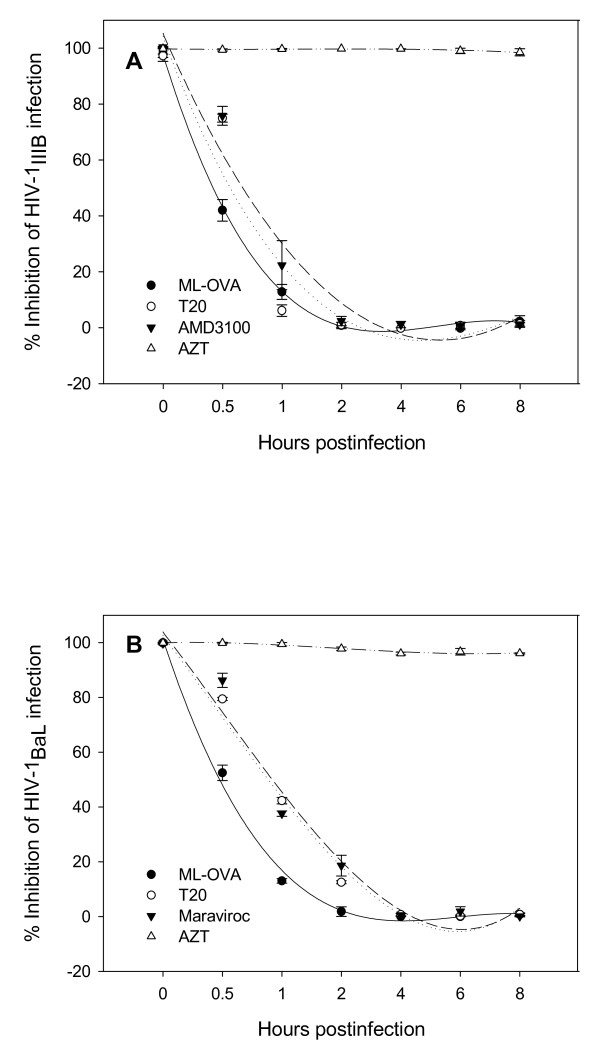
**Time-of-addition assay**. Inhibition of infection by HIV-1_IIIB _(A) and HIV-1_BaL _(B) by ML-OVA and the control compounds when added at different intervals post-infection was tested using a time-of-addition assay. Each sample was tested in triplicate, the experiment was repeated twice, and the data are presented in means ± SD.

ML-OVA was shown to inhibit HIV-1 Env-mediated cell-cell fusion (Table 2), suggesting that it may inhibit HIV-1 infection by blocking HIV-1 entry. Here we performed a single-round entry assay using HIV-1_NL4-3 _virions and TZM-bl cells. The results showed that ML-OVA, HP-OVA, and SU-OVA, all inhibited single-round virus entry, while the unmodified OVA had no such activity (Fig. 4). ML-OVA could not block the single round entry of the VSV-G pseudovirus (data not shown), suggesting that ML-OVA may specifically target HIV-1 at the entry stage. To determine whether ML-OVA could also act at the late stage of the HIV-1 replication, we carried out a time-of-addition assay using both X4 and R5 HIV-1 strains and the well-know HIV-1 entry/fusion inhibitors and RTI as controls. As shown in Fig. 5, the nucleoside reverse transcriptase inhibitor (NRTI) - AZT exhibited potent anti-HIV-1 activity against both X4 virus HIV-1_IIIB _and R5 virus HIV-1_BaL _when it was added to cells before viral infection and 1 ~8 h post-infection, while the HIV entry inhibitors, such as T20 (against both HIV-1_IIIB _and HIV-1_BaL_), AMD-3100 (against HIV-1_IIIB_) and Maraviroc (against HIV-1_BaL_), exhibited significantly decreased inhibitory activity when they were added 0.5 ~2 h post-infection. ML-OVA showed inhibitory profiles similar to those of HIV entry inhibitors, suggesting that ML-OVA exerts its antiviral action at the early stage of HIV-1 replication.

### ML-OVA bound with cells express HIV-1 Env or CD4

**Figure 6:**
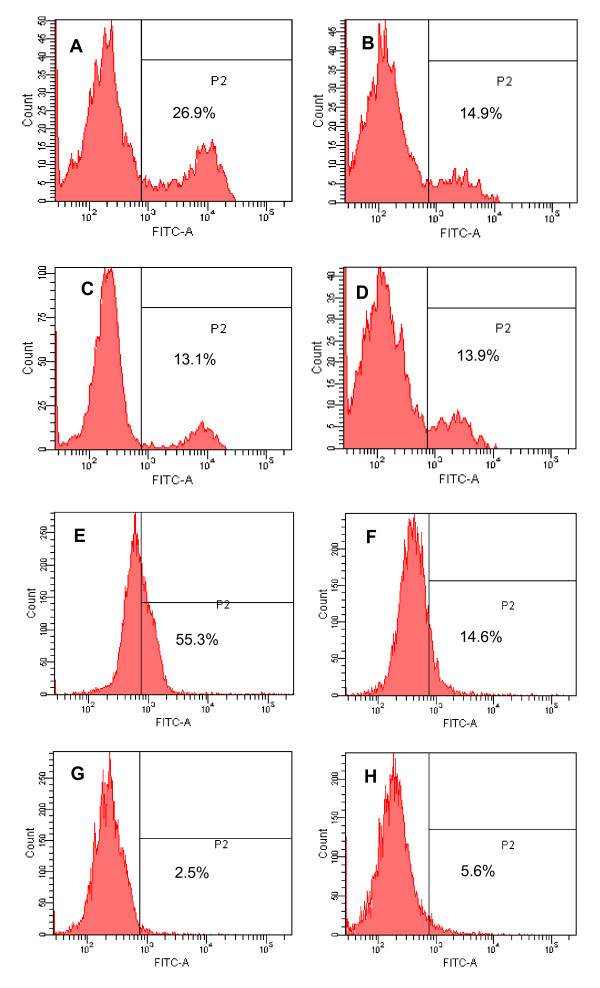
**Flow cytometric analysis of binding of ML-OVA to cells expressing HIV-1 Env or CD4 molecule**. (A) ML-OVA + CHO-WT cells; (B) ML-OVA + CHO-EE cells; (C) OVA + CHO-WT cells; (D) OVA + CHO-EE cells; (E) ML-OVA + HeLa-CD4-LTR-β-gal cells; (F) ML-OVA + HeLa cells; (G) OVA + HeLa-CD4-LTR-β-gal cells; and (H) OVA + HeLa cells.

As mentioned above, ML-OVA is highly effective in inhibiting fusion between the effector and target cells, suggesting that it may interact with either the HIV-1 Env on the effector cells or the CD4 receptor on the target cells. Here we used flow cytometry to analyze the binding activity of ML-OVA to CHO-WT cells that express HIV-1 Env or HeLa-CD4-LTR-β-gal cells that express CD4 molecule, using CHO-EE and HeLa cells that express neither HIV-1 Env nor CD4 as controls. The results showed that ML-OVA could significantly bind with both CHO-WT and HeLa-CD4-LTR-β-gal cells (Fig. 6A, and 6E). However, it had only background binding to CHO-EE and HeLa cells (Fig. 6B and 6F), at the similar level as the unmodified OVA (Fig. 6C, D, G and 6H). These results suggest that ML-OVA is able to interact with both HIV-1 Env and CD4 receptor on cell surfaces.

### ML-OVA bound with both gp120 and CD4 molecules and blocked the gp120-CD4 interaction

The first step of HIV-1 entry into a CD4^+ ^target cell occurs when the surface subunit gp120 of the HIV-1 Env binds to CD4 [39]. Previous study has shown that 3HP-β-LG interfered with the binding of CD4 to HIV and SIV surface Envs as well as monoclonal antibodies specific to the gp120 binding site on CD4 [11]. Using similar approaches, we determined the potential effect of ML-OVA on the interaction between sCD4 and gp120 or gp105, the surface subunits of HIV-1 or HIV-2 Env, respectively. As shown in Table 5, ML-OVA was highly effective in blocking the interaction between sCD4 and gp120 from HIV-1_IIIB_, HIV-1_BaL_, and HIV-1_MN _and between sCD4 and gp105 from HIV-2_ROD_, while unmodified OVA exhibited no inhibition at the concentration up to 100 μM. These results indicate that the inhibition of HIV entry by ML-OVA may be attributed to its inhibitory effect on viral gp120 binding to the CD4 molecule on the target cell.

**Table 5:** Inhibitory activity of ML-OVA on the association between sCD4 and distinct HIV envelope proteins.

Chemically modified protein	Inhibitory activity (μM)^a^	The HIV envelope proteins			
		
		gp120 of HIV-1_IIIB_	gp120 of HIV-1_MN_	gp120 of HIV-1_BaL_	gp105 of HIV-2_ROD_
ML-OVA	IC_50_	0.471 ± 0.063	1.161 ± 0.092	1.397 ± 0.008	0.466 ± 0.076
	IC_90_	3.258 ± 0.413	8.567 ± 2.360	19.77 ± 1.491	11.36 ± 3.721
OVA	IC_50_	>100	>100	>100	>100
	IC_90_	>100	>100	>100	>100

^a^The measurements were performed in triplicate, and the experiment was repeated twice. Data are presented in means ± SD.

**Figure 7:**
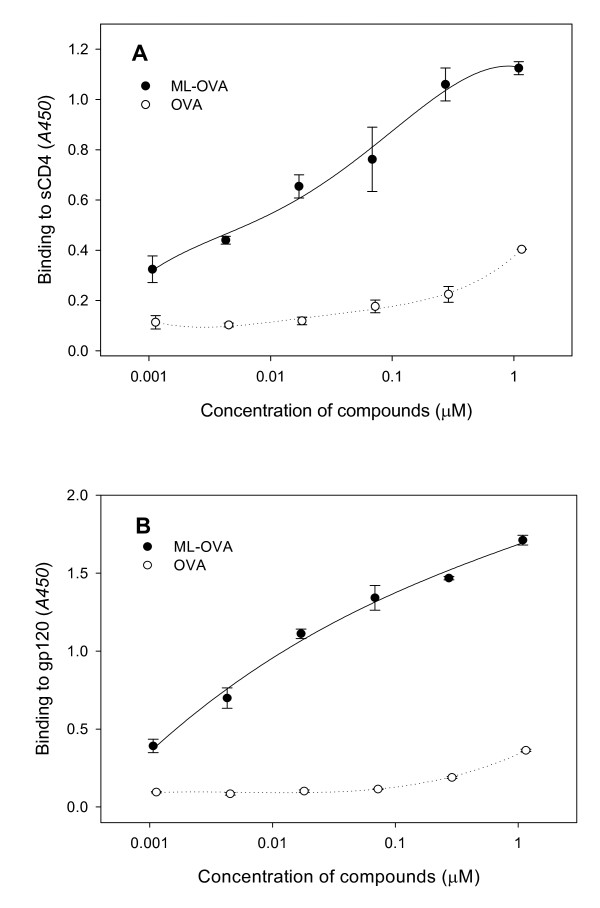
**The binding of ML-OVA to sCD4 and gp120 as assessed by ELISA**. (A) Dose-dependent binding of ML-OVA to sCD4. (B) Dose-dependent binding of ML-OVA to gp120 from HIV-1_IIIB_. Each sample was tested in quadruplicate, the experiment was repeated twice, and the data are presented as means ± SD.

To further characterize the target of ML-OVA, the interaction of ML-OVA with gp120 or sCD4 was examined by ELISA. The results showed that the interaction of sCD4 (Fig. 7A) and gp120 from HIV-1_IIIB _(Fig. 7B) bound with ML-OVA in a dose-dependent manner. Unmodified OVA exhibited no significant binding effects at the concentration up to 1 μM. From the OD_450 _values of the binding assays, ML-OVA bound with gp120 more efficiently than with CD4. These results indicate that the targets of ML-OVA are both on gp120 and CD4, especially gp120.

### ML-OVA was resistant to trypsin hydrolysis

**Figure 8:**
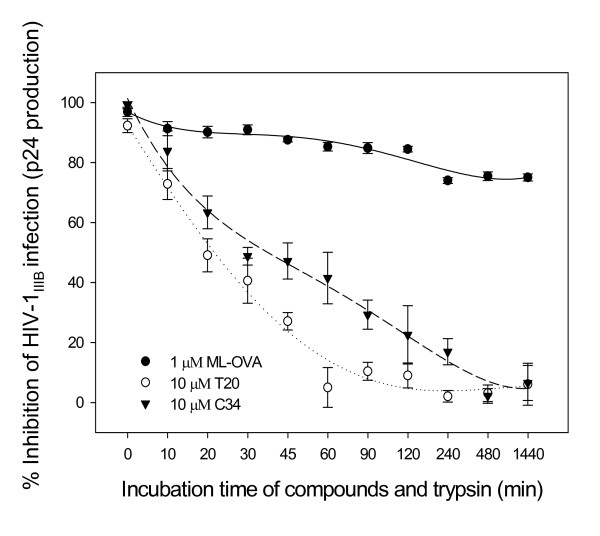
**Sensitivity of ML-OVA to digestion by trypsin**. The remaining anti-HIV-1_IIIB _activity of ML-OVA, T20 and C34 after incubation with trypsin for varying intervals of time was determined by ELISA for p24 antigen production. All the samples were tested in triplicate, the experiment was repeated twice, and data are presented in means ± SD.

Trypsin is one of the principal digestive proteases in the human body, especially in the vaginal flora, which predominantly hydrolyzes proteins/peptides at the carboxyl side of arginine and lysine residues. Since most lysine and arginine residues in OVA had been modified by ML, we intended to know whether ML-OVA is susceptible to trypsin hydrolysis by measuring the anti-HIV-1_IIIB _activity of ML-OVA treated with trypsin. As shown in Fig. 8, ML-OVA retained more than 80% of its anti-HIV-1 activity even 24 h after its incubating with trypsin beads, while the peptidic HIV-1 fusion inhibitors, C34 and T20, lost most of their antiviral activities 2 h post-treatment with trypsin. These results indicate that ML-modified ovalbumin become resistant to trypsin hydrolysis.

### SF and VFS had no significant effect on the anti-HIV-1 activity of ML-OVA

**Figure 9:**
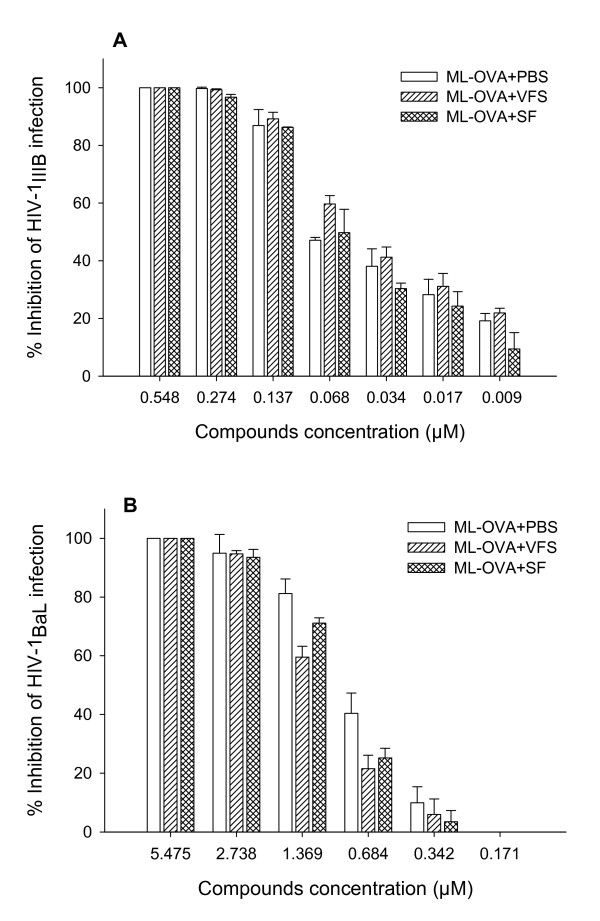
**The effect of human SF and VSF on the anti-HIV-1 activity of ML-OVA**. The antiviral activities against HIV-1_IIIB _(A) and HIV-1_BaL _(B) in the presence or absence of SF and VSF were assessed using p24 assay as described in the Materials and Methods. The inhibitory activity was detected by an ELISA assay. Each sample was tested in triplicate, and the data are presented as means ± SD.

Human body fluids such as seminal and vaginal fluids may have negative effect on the efficacy of the topical microbicides [30,31,40], while sexual transmission of HIV occurs in presence of those human body fluids. Therefore, it is necessary to determine the potential effect of SF and VFS on the anti-HIV activity of ML-OVA. As shown in Fig. 9, neither SF nor VFS had significant effect on the inhibitory activity of ML-OVA against infection by HIV-1 X4 and R5 strains. The IC_50 _values of ML-OVA for inhibiting HIV-1_IIIB _infection in the presence of SF and VFS were 0.045 μM and 0.030 μM, respectively, while that of PBS control is 0.031 μM. The IC_50 _values of ML-OVA for inhibiting HIV-1_BaL _infection in the presence of SF and VFS were 1.029 μM and 1.033 μM, respectively, whereas that of PBS control is 0.769 μM. Those results suggest that SF and VFS have no negative effect on the application of ML-OVA as a microbicide.

## Discussion

In the present study, we screened for ideal chemically modified agents as potential microbicides, and five non-bovine-origin proteins were used in our studies. First, these agents were modified by one anhydride, 3-hydroxyphthalic anhydride (HP). By evaluating their anti-HIV-1 activities against lab-adapted X4 and R5 viruses, it was revealed that some common proteins, such as OVA, RSA and PSA, could be converted into effective anti-HIV inhibitors by modification of their positive residues (lysine and arginine) with 3HP (Table 1). On the other hand, HP-modified proteins from gelatins displayed very low anti-HIV-1 activity with uncharacteristically high percentages of lysine modification. By analyzing the structure of the proteins found to possess antiviral activity, OVA, RSA and PSA were found to have representative globulins identical to bovine β-lactoglobulin. By contrast, the gelatins used in this study are derived from collagens, which had different structure and conformation. The absence of anti-HIV activities of these modified proteins indicated that HIV blocking abilities might not be solely dependent on the modified lysine or arginine but also on the protein conformation. Thus, the presence of specific globular structures might play an important role in the anti-HIV activity of OVA, RSA and PSA.

Although both RSA and PSA exhibited anti-HIV-1 activity similar to OVA after modification with HP, we selected OVA for further studies. Ovalbumin is the main protein found in egg white with a molecular weight of about 43 kd by SDS-PAGE. It is made up of 385 amino acids, containing 20 lysine (5.19%) and 15 arginine (3.90%) residues [41,42]. Most of these positively charged amino acids in the protein have been modified by anhydrides, which could convert the proteins into anti-HIV inhibitors (Table 1). Importantly, because OVA is easily isolated from chicken eggs, it is much more economical than albumins purified from animal sera. Furthermore, the products from sera have the added risk of contamination by infectious pathogens. Since an ideal microbicide should be inexpensive and safe, chicken OVA may be the most suitable protein for modification as an anti-HIV agent to prevent HIV sexual transmission.

OVA is a common antigen used in the immunogenicity studies. One may raise a concern about the potential of ML-OVA to induce harmful immune responses in vaginal mucosa when it is used as a topical microbicide. However, a number of studies have showed that mucosal immunization through intravaginal and intrarectal administration with soluble proteins, including OVA, in absence of adjuvants, are usually unable to induce strong local immune responses [43-45]. Therefore, intravaginal or intrarectal application of ML-OVA as a microbicide may not be expected to elicit harmful local immune responses. Another problem for the development of chemically modified OVA as a topical microbicide is the potential risk of causing side effect in people who are allergy to egg protein [46]. But fortunately, egg allergy occurs seldomly in adults, but mostly in young children (less than 5 years old) [47]. Therefore, we expect that there will be only very few adults with egg allergy, and those people should be excluded from the clinical trials of ML-OVA-based microbicide.

Although HP-OVA is a potent anti-HIV agent, the phthalate derivatives were reported to have carcinogenic potential [35-38]. Therefore, the use of HP-OVA as a microbicide for the prevention of HIV-1 sexual transmission raises safety questions. To search for alternate anhydrides as chemical modifiers of OVA, we selected two other anhydrides, succinic anhydride (SU) and maleic anhydride (ML), for the chemical modification. SU is one of the food additives or pharmaceutical excipients. Carcinogenesis studies of SU in B6C3F1 mice and F344/N rats performed by National Toxicology Program showed that SU had no carcinogenic activity [48]. ML is also a common anhydride used in pharmaceuticals. A maleic anhydride-divinyl ether copolymer (MVE-2) was shown to inhibit mammary and urinary bladder carcinogenesis [49].

All three anhydrides (SU, ML and HP) were sufficiently potent to convert OVA into an effective anti-HIV agent. The percentages of modified and unmodified lysine and arginine residues were dependent on the concentration of anhydrides and pH of the reaction system with the strength of anti-HIV activity correlated to the successive increase of positively charged residues. These results were consistent with our previous studies with 3HP-β-LG [6,7]. Therefore, the optimal condition to produce potent anti-HIV modified OVAs, as suggested from this study, is 40 mM anhydride used at pH 8.5 for 20 mg/ml OVA. Under this condition, ML-OVA demonstrated more efficacy than SU-OVA in blocking HIV-1 infection, especially the sexually transmitted R5 virus. Furthermore, a series of poly [styrene-*alt*-(maleic anhydride)] derivatives were reported as potential microbicide candidates with high efficacy and low cytotoxicity [50]. Therefore, we selected ML-OVA for further investigation.

ML-OVA displayed broad antiviral activities against HIV-1, HIV-2 and SIV with low cytotoxicity. While ML-OVA is less potent against laboratory-adapted R5 BaL strain than X4 strains, it is effective in inhibiting the infection of primary R5 viruses with distinct genotypes and phenotypes (Table 4). Interestingly, ML-OVA was shown to be effective against the HIV-1 variants resistant to AZT and T20 (Table 4), suggesting that ML-OVA is capable of preventing the sexual transmission of HIV-1 strains that are resistant to the currently used antiretroviral therapeutics. In addition, ML-OVA is effective in inhibiting HIV-2 infection, suggesting that this microbicide candidate may also be applicable in West Africa where HIV-2 is prominent. Our studies also showed that ML-OVA could potently inhibit infection by SHIV_SF162 _(R5), SHIV_SF33A _(X4) and SIV with IC_50 _ranging from 0.189 to 1.312 μM (Table 4). Since both SHIV and SIV can be used for infection of rhesus macaques, ML-OVA will be tested in a non-human primate model for evaluation of its *in vivo *efficacy against SHIV or SIV infection through vaginal challenge.

A microbicide capable of inhibiting HIV infection by targeting the entry step has the obvious advantage of blocking HIV transmission at the outset of viral infection [51]. By using cell-cell fusion assay, single round viral entry assay, and time-of-addition assay, we demonstrated that ML-OVA, like 3HP-β-LG, inhibits HIV-1 infection by targeting the early stage of viral replication, particularly the viral entry/fusion processes. Subsequent studies suggested that ML-OVA bound with the cells expressing HIV-1 Env and CD4 (Fig. 6). Using ELISA, we demonstrated that ML-OVA could bind to both gp120 and CD4 molecules, with higher binding efficiency to the former (Fig. 7) and it was effective in blocking the binding of gp120 to sCD4 (Table 5). The binding ability with both gp120 and CD4 may arise from the negatively-charged residues of ML-OVA. Consistently, several negatively-charged polymeric microbicide candidates, such as cellulose acetate phthalate (CAP), carrageenan, cellulose sulfate, PRO-2000, and dextran sulfate, can also interact with gp120 and CD4 to block HIV-1 entry. The positively-charged side chains of lysine and arginine residues of OVA were converted to negatively-charged side chains after modification by anhydride. It is the chemical structure of anhydrates that accounts for the effect of different anhydride OVA modifications on HIV inhibitory activities. Specifically, the only difference between maleic and succinic anhydride was the double bond between C3 and C4 in maleic anhydride, which led to the stronger inhibition abilities of ML-OVA over those of SU-OVA on HIV infection. 3-hydroxyphthalic anhydride has a hydrophobic aromatic group, leading to the most potent anti-HIV activity. These findings suggest that the aromatic and unsaturated structure in anhydrides might contribute to the difference in antiviral activities of these modified OVAs.

The disadvantage of protein/peptide drugs, such as T20, is their short half life resulting from the hydrolysis by proteases like trypsin. Since trypsin is the major protease in human and predominantly cleaves peptide chains at the carboxyl side of the amino acids lysine and arginine in human beings, we tested whether ML-modified proteins are sensitive to trypsin. Notably, treatment of ML-OVA with trypsin did not affect its anti-HIV-1 activity, indicating that the ML-modified lysine and arginine residues became resistant to trypsin.

An ideal microbicide candidate should be active against HIV-1 infection in the presence of human body fluids, such as seminal fluid or cervicovaginal fluid, because the topical microbicides will be applied intravaginally or intrarectally. In the present study, we tested the effects of seminal fluid and vaginal fluid simulant on anti-HIV activities of ML-OVA. The results indicate that the antiviral activities of ML-OVA are stable in the presence of those fluids (Fig. 9), suggesting that ML-OVA should be active being used as a microbicide.

Currently, there is less enthusiasm for developing polyanionic anti-HIV microbicides due to the failure of clinical trials of several polyanionic polymer-based microbicide candidates, including cellulose sulfate (Ushercell) [52], Carrageenan (Carraguard) [53], and PRO 2000 [54-56], because they have much lower antiviral activity against primary R5 HIV-1 isolates than laboratory-adapted X4 viruses [57,58]. However, we believe that ML-OVA has better potential than those polyanionic polymers for microbicide development because our studies have shown that ML-OVA exhibit highly potent antiviral activity against a broad spectrum of primary R5 HIV-1 isolates (Table 4). Furthermore, ML-OVA can be used in combination with a nonnucleoside reverse transcriptase inhibitor (NNRTI) as a combination microbicide for prevention of infection by HIV-1 strains with resistance to reverse transcriptase inhibitors (RTIs) since our studies have shown that ML-OVA is highly effective against RTI-resistant variants (Table 4). Most recently, Fang, *et al*. identified a series of poly [styrene-*alt*-(maleic anhydride)] derivatives with much more potent antiviral activity against both R5 and X4 HIV-1 strains than cellulose sulfate, Carraguard and PRO 2000 [50], which is an example of developing polyanionic polymers with improved anti-HIV-1 efficacy as new microbicide candidates.

## Conclusion

In general, the present study provides the optimal conditions (40 mM of anhydrides at pH 8.5) for large-scale production of anhydride OVAs. By evaluating the anti-HIV activities and analyzing the mechanism of action, we conclude that such modified OVAs are broad-spectrum HIV entry/fusion inhibitors through blocking viral entry. By its broad antiviral potency, resistance to trypsin hydrolysis, easy preparation, low production costs, wide availability and absence of carcinogenic phthalic group, ML-OVA has promising potential to be developed as an anti-HIV microbicide for preventing HIV sexual transmission.

## Competing interests

The authors declare that they have no competing interests.

## Authors' contributions

SL and SJ conceived the idea and designed research. LL, PQ, JY, LL, ST, HL, XZ and XC performed research. LL, SL, and SJ analyzed the data and wrote the paper. SW critically reviewed and edited the paper. All authors read and approved the final manuscript.
